# Care-receivers with physical disabilities’ perceptions on having humanoid assistive robots as assistants: a qualitative study

**DOI:** 10.1186/s12913-024-10857-9

**Published:** 2024-04-25

**Authors:** Linda Sørensen, Dag Tomas Johannesen, Helinä Melkas, Hege Mari Johnsen

**Affiliations:** 1 University of Agder, Kristiansand, Norway; 2 Lappeenranta-Lahti University of Technology LUT, Lappeenranta, Finland

**Keywords:** Assistive technologies, Assistive robots, Independence, Autonomy

## Abstract

**Background:**

People with physical disabilities due to disease or injury face barriers to their daily activities and participation in society. Many depend on formal or informal caregivers for assistance to live independently. However, future healthcare challenges due to demographic changes threaten access to home care and assistants. Assistive technologies, such as robots for physical assistance, can support the independence and autonomy of people with physical disabilities. This study explore Norwegian care-receivers’ perceptions of using robot assistance in their homes, including preferences for tasks acceptable or unacceptable for robot assistance and the underlying reasons.

**Method:**

Purposive sampling was employed to recruit 18 participants, aged between 18 and 77 years, with differences in physical function including diagnoses such as stroke, spinal cord injury, amputations, and muscular dystrophy. Qualitative data were gathered through four focus group interviews wherein participants watched videos featuring a humanoid assistive robot, EVEr3. The collected data underwent analysis using reflexive thematic analysis.

**Results:**

Three themes with associated sub-themes were constructed: (a) How a robot could assist in daily life, (b) The robot’s appearance and functionality, and (c) Concerns about having a robot as an assistant. The participants welcomed the idea of a future robotic assistant in areas that may contribute to an increased feeling of independence and autonomy.

**Conclusion:**

A robot assisting in activities of daily living would need to be individually customized to meet the needs of each user in terms of which tasks to assist with, how to assist in these defined tasks, and how it is controlled.

**Supplementary Information:**

The online version contains supplementary material available at 10.1186/s12913-024-10857-9.

## Background

According to the World Health Organization (WHO) more than 15% of the global population lives with a form of disability [1]. Physical disability is characterized by a loss of function caused by either disease (resulting from harm to organs [2]) or injury (stemming from accidents or attacks). Examples encompass individuals with conditions such as cerebral palsy, muscular dystrophies, stroke, and spinal cord injuries [3]. Persons with physical disabilities face significant barriers in many aspects of civil society, such as poor physical accessibility, transport issues, and social obstacles, preventing them from fully participating on an equal footing [4–6].

Many people with reduced physical function require assistance from both formal and informal caregivers to participate in activities of daily living. In Norway, with a population of 5,5 million [7], 166,600 people in total, were care receivers of home-based services from municipalities, and 10,200 caregivers received support for caring for their relatives in 2019. Since 1994, the number of younger people (under 67) receiving home care assistance has tripled [8]. While most users report satisfaction with the services they receive, younger age groups have reported dissatisfaction with the flexibility and availability of the services [9, 10].

### Demographic challenges

By 2030, there will be an estimated shortage of 5.7 million nurses worldwide [11]. This shortage is partly due to demographic factors such as population growth and aging and to the ambition of service delivery within the context of the Sustainable Development Goals [11, 12]. Like other countries, Norway is facing significant challenges in accessing adequate and competent personnel, which may reduce the ability to offer assistance with activities of daily living to people with disabilities, affecting their independence and quality of life [13]. In the literature, independence is often synonymously associated with autonomy [14, 15].

The concept of individual autonomy is described as the ability to act freely in accordance with a self-chosen plan [16]. Within health services, strengthening an individual’s capacity and ability to control factors that affect their health and lifestyle is also referred to as empowerment by the WHO (2006).

### Assistive technologies

Assistive technologies, such as wheelchairs, hearing aids, and prostheses, aim to provide services that support autonomy and a sense of independence and promote inclusion and participation for people with disabilities. These technologies can enable people to live healthy, productive, independent, and dignified lives and participate in education, the labor market, and civil life [17]. As a form of assistive technology, physical robots can assist users with physical disabilities with tasks such as eating, showering, or grasping objects. Examples of commercially available assistive robots that can assist users in gripping objects are Jaco® and iARm, which are often placed on the user’s electric wheelchair. In addition, Neater Eater is a robot assisting the user with reduced arm function in eating (placed on the user’s table). Robotic showers and intelligent tables (positioning itself after the user’s command) are currently being tested, but so far, they seem not to have reached the commercial market [18–21].

### Assistive robots

Additionally, the scientific robotics community is exploring solutions for so-called social robots that can adapt to the “local” rules of social conduct, understand their human companions’ current moods and wishes, and learn by copying humans. These robots can be controlled from a distance (i.e. Home-services), by the user or be fully automized to provide services. Socially assistive robots may provide social companionship and supervise simple exercises [22].

Few robots can provide users with various physical assistance, such as simple carry and delivery tasks. Examples of such robots include Care-O-bot, TIAGo, Lio, and the Hobbit [21, 23–25], yet presently, these robots are primarily perceived as research platforms.

Assistive robots have the potential to contribute to users with disabilities being more independent. However, a person’s decision to use robot technologies is determined by how users accept these technologies [26, 27]. According to Venkatesh et al., [26] whose Unified Theory of Acceptance and Use of Technology (UTAUT) focuses on IT systems, the intention to use a technology, such as robotic systems, can be predicted by factors such as whether potential users believe using the technology would enhance their performance and whether they believe using the technology would be relatively free of effort. Heerink et al. [27], whose work centers on social robots supports these findings. Therefore, exploring users’ perceptions of usefulness and ease of use is essential to predict the future adoption of robot technologies.

Researchers have primarily investigated older users’ experiences interacting with assistive robots [25, 28, 29]. Studies suggest that older users find these robots enjoyable, friendly, and safe [30, 31]. However in the studies by Wu et al., [32] and Lee et al. [33], some participants who do not have functional decline express that the robots are irrelevant to them. The area of assistive robots for physically disabled individuals (young or old) is still in its early stages. Therefore, this study aimed to explore physically disabled home service users’ perceptions of robotic assistance, including which assistance tasks they would accept to be delivered by a robot.

**Fig. 1:**
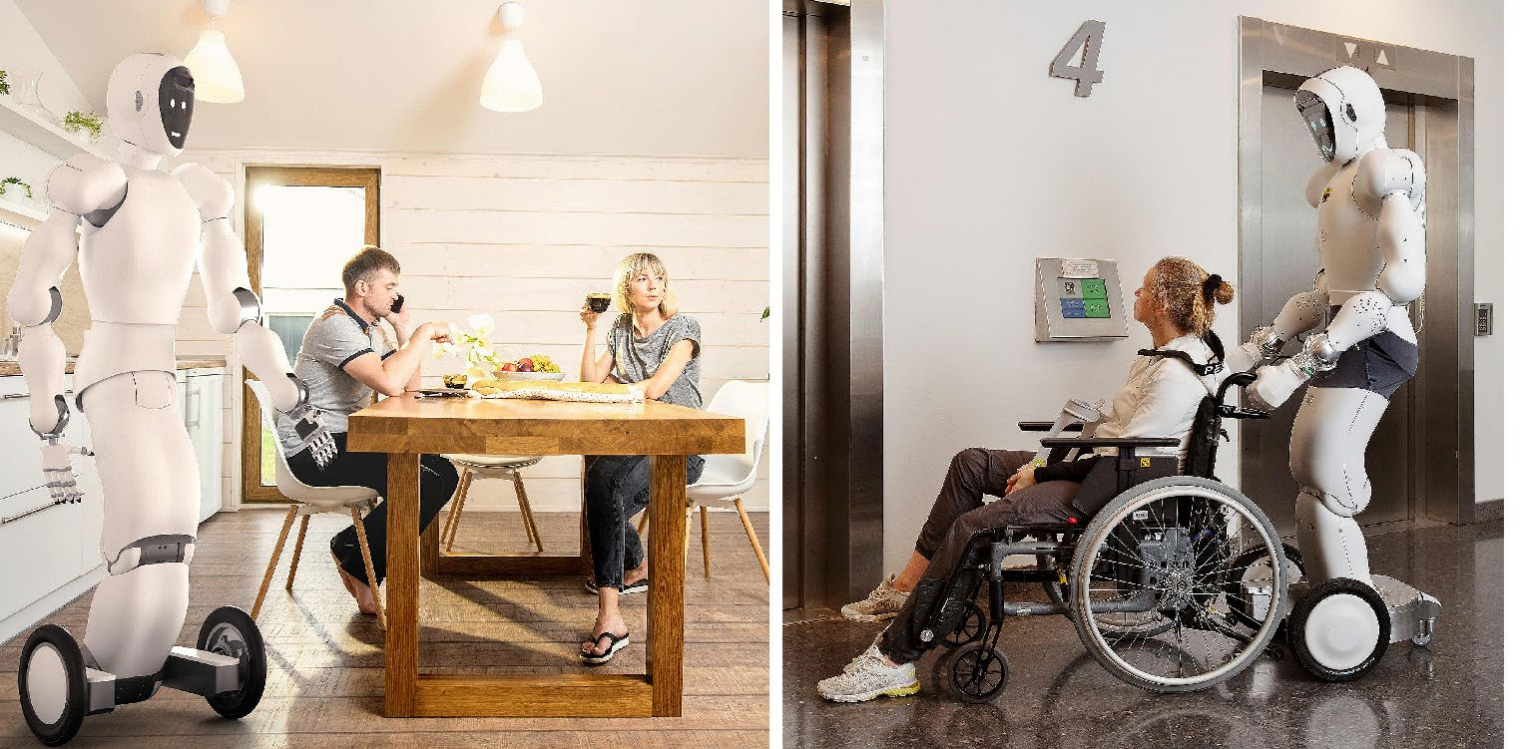
EVEr3, the example robot used to illustrate assistive robots for physical assistance [41]

The study is part of a project investigating humanoid robotics for healthcare at the University of Agder, Norway. The university has acquired a humanoid robot named EVEr3 (EVE) (Fig. 1) for research purposes. In this study, EVE was an example of an assistive robot for the participants, who had no prior knowledge or experience with assistive robotics. The project has two parts. This paper, which reports the result of the first study, used films of EVE to investigate participants’ needs and acceptance. The second study will use results of this study to develop the robot for personalized tasks and will investigate usefulness and usability in interaction with the robot.

Our research questions for this study were:

- Which tasks would home care receivers with physical disabilities accept or not accept to be delivered by an assistive robot, exemplified by the robot EVE?

- What are physically disabled home care receivers’ perceptions of receiving robotic assistance?

## Methods

### Design and method

This study used an experiential, descriptive, and interpretive qualitative design [34] and employed focus group interviews [35] as the data collection method. This design is suitable for investigating people’s perspectives [36, 37].

### Recruitment of participants

The study’s inclusion criteria consisted of individuals aged 16 to 70 with a physical disability who resided at home, encompassing various settings such as supported housing or residential homes, and were receiving formal care from either municipality, home services or personal assistants. Participants needed to be able to communicate in a focus group activity, which excluded individuals with severe cognitive difficulties or aphasia. Assisted communication was not an exclusion criterion.

Participants were recruited through purposive sampling via coordinators of health services at a rehabilitation hospital in the eastern region of Norway and a municipality in the south region of Norway. The coordinators suggested relevant candidates to the researcher based on the inclusion/exclusion criteria. We aimed at recruiting approximately 25 participants. Coordinators invited 37 candidates to participate and provided information about the project [35]. The recruitment process persisted until the gathered data was deemed comprehensive enough to serve as a solid foundation for addressing the research questions [38]. Seven participants were unable to participate on the specified dates. Three declined to attend without giving any reason. After inclusion, three participants withdrew for health reasons, one withdrew due to work, and three withdrew due to other appointments. During the summer of 2022, five focus group interviews including 20 participants, were conducted with 2–5 participants per group (No. 1: 5 participants, No. 2: 5, No. 3: 5, No. 4: 3, No. 5: 2). The researchers decided to exclude the data from the fifth group because it had only two participants, and one had language difficulties (Aphasia), which violated the communicated exclusion criteria.

### Contextual background and participants’ level of care

The Norwegian healthcare system is organized on three levels: national, regional, and local. The Law on Municipalities and Care Services [39] provide general descriptions of the services that are the responsibility of the municipalities (local level). However, each municipality decides how best to serve its population with its services, such as home nursing or assistants.

Users with comprehensive needs living at home are entitled to primary care (home nursing). Individuals under the age of 67 can apply for “user-controlled personal assistance” (UPA) [40]. UPA is personal and practical assistance provided by assistants of the user’s choice. UPA aims to ensure equal participation in society for individuals with disabilities.

This study took place in two health regions in Norway. Eleven of our participants had UPAs, two had their applications for UPA rejected by the municipality, and two were still waiting for replies to their applications. Ten of our participants received care from home services, some in combination with UPA services. All participants received care from informal caregivers, such as spouses, parents, and siblings.

### Data collection

The interview guide were developed by the research team, based on the aims of this study, findings from literature reviews [25, 30], and literature on technology acceptance [27]. The semi-structured interview included open-ended questions and allowed for follow up questions and free dialog in the groups. Two healthcare employees familiar with the patient groups and a user consultant with reduced physical function (spinal cord injury, wheelchair user) tested the interview guide.

Four focus group interviews took place in meeting rooms at a rehabilitation hospital and a university, prioritizing availability, comfort, and convenience when choosing locations. Lunch or a light snack was provided. The focus group interview consisted of a two-part semi-structured interview, following the recommendations for structure by Krueger and Casey [35].

The group was led by the first author and assisted by the last author, both having experience conducting focus group interviews. The first author is an occupational therapist with prior experience working with patients with similar disabilities as the group participants, and the last author is an experienced intensive care nurse. None of the researchers were clinically engaged with the participants; however, one had affiliations with the clinic where some participants had received treatment. The first and the last author assisted the participants during the interview, such as providing drinks or helping the interviewees with a snack or attending to acute health-related issues if they occurred. Half of the participants were accompanied by their assistants, who were asked to sit outside during the interview.

The sessions began with repeating the initial information about the project, which the participants had received prior to attending, including reminders of their right to withdraw and the mutual confidentiality between participants in the group. The participants then completed a short questionnaire on their type of disability, tasks they needed assistance with, and who assisted them.

After serving lunch or a light snack (depending on the time of the interview), the interview started with a round where the participants introduced themselves. The interview continued by covering two main topics. The first topic was related to the kind of help the participants needed, the assistance they were provided with, and how well the assistance fulfilled their needs. Halfway through the interview, the participants were shown three short films of the example robot EVE [41] and were informed about this robot’s capabilities. The videos showed the humanoid robot in different situations, such as security, placing groceries, and cooking, not specifically related to health or caring tasks [42]. The second topic for the interview concerned the prospect of having a robot assist in the tasks the participants had identified as necessary in the initial questionnaire and the first part of the interview. Participants were asked about their perceptions of having a robot to assist in the aforementioned tasks in their homes and how they believed it would affect their lives. (Additional file 1: Interview guide). Throughout the interview, participants demonstrated a dynamic engagement with the study’s objectives. The sessions unfolded as a nonlinear communication characterized by participants seamlessly navigating between discussions about their needs and potential interactions with robots, extending beyond the example robot provided. This fluidity underscored the participants’ awareness of the study’s purpose, showcasing their ability to intricately interweave their thoughts on both subjects. By the end of the interview participants were asked to verify summary comments made by the moderators.

One of the focus group interviews was conducted on a secure video conference platform (join.nhn.no) because those participants could not travel to the physical locations. This video conference interview was conducted similarly to the other four interviews. Participants were given information about the project, and informed consent was obtained before the video conference interview. Before the video conference interview, participants were provided links to the three films about the robot and were prompted to watch them.

The interviews, including the video conference interview, lasted between 60 and 90 min and were recorded on two digital recorders. The recorded files were transferred to a secure storage area and then deleted from the recorders to ensure confidentiality.

### The example robot

The example robot EVE, is a full-size humanoid robot developed for research purposes. It is wireless, 180 cm tall, weighs 80 kg, and moves around on three wheels. It has a face with “eyes” and a smile. The face can be changed in accordance with a user’s preferences. It has five fingers and can manipulate smaller and larger objects, such as picking up a bottle of water, retrieving something from the floor, or opening doors.

Currently, the robot is being used for research, mainly controlled by Virtual Reality glasses and controllers. EVE can navigate autonomously and is being developed to perform relevant actions and respond to voice commands [41]. The robot served as a model in the study; nevertheless, participants were encouraged not to restrict their feedback to this specific robot. All comments on robots, even those unrelated to the provided knowledge on EVE, were welcomed.

### Ethics approval and consent to participate

All methods were carried out in accordance with relevant guidelines (Helsinki Declaration) and national law. The study was approved according to the Research Ethics Committee at the Regional Committees for Medical and Health Research Ethics (467937) and the University’s ethical board. All participants gave their written informed consent to participate in the study.

### Analysis

All interviews were transcribed verbatim by the first author, omitting minor speech hesitations to improve readability. To ensure confidentiality and anonymity, identifiable details were anonymized, and participants and other mentioned persons were assigned numbers. A six-phase reflexive thematic analysis, developed by Braun and Clarke [34], was used to explore the patterns and complexity in the participants’ statements. Initially, the researchers became familiar with the dataset (Phase 1) before coding the data with NVivo software (Phase 2). The coding was revised several times before clustering codes to determine evident patterns and generate initial themes (Phase 3). Three themes were developed by refining and naming the initial themes (Phases 4 and 5) before writing the report (Phase 6) [34]. The first author conducted the coding and analysis process, which was reviewed and discussed by all authors to expand understanding and provide clarity when defining the final themes.

The reflexive thematic analysis [43] method distinguishes itself from other analytical approaches by focusing on identifying patterns within qualitative data. A theme in this method captures a significant aspect of the data relevant to the research question, representing a structured response or meaning within the dataset. These patterns may not necessarily reflect the frequency of statements but should contribute to a coherent and internally consistent narrative. Consistent with Braun and Clarke’s approach (2006), our aim was to delve deeper than simply paraphrasing data extracts. We emphasized not only uncovering the ‘story’ within each theme but also understanding how it contributes to the broader narrative of the data in relation to the research question. An example of the analysis process is presented in Table 1.

**Table 1:** Example of the analyzing process

Participant statement	Code	Sub-theme	Themes
*“I imagine that it [a robot] uses its arm, and then I hang on the arm, yeah, just lifts me, and that’s how I imagine it, and then he [a robot] goes over to the chair and then like gently puts med down […]. That would be nice”.*	Transfers	*The potential for robots as enabling technologies*	How a robot could assist in daily life

The main author maintained a reflexive journal throughout the analysis as part of the systematic and transparent process. To answer the first research question about which assistance tasks the participants would accept or reject assistance from a robot, the researchers utilized the International Classification of Function, Disability and Health (ICF) to organize the findings. ICF is a framework developed by the World Health Organization (WHO) for measuring health and disability at both individual and population levels. Other authors have previously used ICF concerning assistive robotics to evaluate assistive technologies’ impact on users’ lives and group activities that humans perform [44, 45].

## Results

### Participants

The final dataset included five female and 13 male participants between 18 and 77, with a median age of 53 years. Table 2  shows their sex, age, diagnosis, and the assistance they receive in daily activities.

The participants exhibited diverse diagnoses and varying degrees of assistance requirements. Our primary focus, however, was not centered around their specific diagnoses but rather on their functional abilities and the extent of assistance necessary for engaging in ADL.

Among the participants, three individuals experienced paralysis in both legs and arms, requiring assistance exceeding 10 h daily, provided by hired caregivers, as well as support from family and friends. The majority of the participants (13 participants) experiencing paralysis in arms and/or legs, who also received hired assistance, supplemented by aid from family and friends, had a higher level of independence during periods of the day. Two participants indicated that they maintained significant independence but needed intermittent assistance during the week.

All participants resided in private homes or apartments, whether owned or rented. Seven lived independently, two resided with their parents, and nine were living with their partners.

Most of the participants had personal assistants (UPAs). Some also had home nursing, and all participants received assistance from informal caregivers such as spouses or friends (Table 2).

**Table 2:** Participants

Participant	Sex	Age	Diagnosis	Assistance	Assistance from			
					Home- nurse	Assistant	Next of kin	Others
F1-1	F	76	SCI-C5	A	x	x	x	
F1-2	F	23	SCI-C5	A	x	x	x	
F1-3	M	26	SCI-C6	A	x	x	x	
F1-4	M	43	Amputation	B	x		x	
F1-5	M	18	SCI-C6	B		x	x	
F2-1	M	56	SCI-T5	B		x	x	
F2-2	M	60	SCI-C3	A				Rehab-center staff
F2-3	M	56	SCI-C4	A	x	x	x	
F2-4	M	77	SCI-T5	C	x		x	
F2-5	F	33	SCI-C5	D		x	x	
F3-1	M	30	SCI-C5	B	x	x		
F3-2	F	59	Muscular dystrophy	A		x	x	Friends
F3-3	M	58	SCI-C4	A		x		
F3-4	M	36	SCI-C6	B		x	x	Friends
F3-5	M	51	SCI-T9	D				Cleaner
F4-1	M	59	Stroke	B	x		x	
F4-2	M	43	Stroke	B	x		x	
F4-3	F	59	Stroke	A	x		x	

Abbreviations: SCI-C: Cervical Spinal Cord Injury, paralysis in arms and legs. SCI-T: Thoracal Spinal Cord Injury, paralysis from waist and down. Assistance categories receive assistance A: more than five times a day, B: 1–3 times a day, C: 3–5 times a day, D: 1–6 times a week

### Results from the analysis

To address the research questions regarding (1) the specific tasks that participants were willing and unwilling to receive robotic assistance with and (2) their perceptions of humanoid robotic assistance in their homes, we identified three themes with associated sub-themes. These were: a) How a robot could assist in daily life, (i) the potential for robots as enabling technologies, (ii) the freedom of being alone, and (iii) the ability to decide what to do and when to do it, b) Appearance and functionality of robots, and c) Concerns about having a robot as an assistant.

### How a robot could assist in daily life

#### The potential for robots as enabling technologies

This theme highlights the participants’ perceptions of how a robot (robots in general) could assist to become more independent in their daily life. The participants expressed 23 tasks, organized according to the ICF format, and presented in Table 3. Participants who reported requiring assistance in both practical and household tasks, and receiving the highest amount of care hours, expressed a greater willingness to delegate various tasks to a robot.

**Table 3:** Need and acceptance of robot assistance

Need and accept	Need, not accept	ICF domain
Scratch the participant Shower assistance Fine motor assistance **Assist in exercising** Walking partner (support walking-exercise)	Skincare Shower assistance Toileting/catheterization Positioning	Body-functions and structures
Prepare/make food **Bring water/food** Clean up after a meal Feeding **Pick up things from the floor** Prepare clothes **Assist in transfers** Assists with PC, cord, plugs Turn on TV **Housework (dusting, tidying up)** Laundry Make/change the bed **Shovel snow** Car maintenance Maintenance of technical aids Answer the door Walking partner	Prepare/make food Assist in transfers	Activities
Prepare for guests Assist at work Activities related to board and organizational work	Assist with grandchildren	Participation

The tasks mentioned by participants were mainly related to practical tasks around the house, such as daily housework and regularly keeping the house tidy. They specifically mentioned tasks such as making the bed, dusting, cleaning the house, and doing laundry. While most participants had arranged for some form of housekeeping, either through their assistants or privately hired help, some were open to using a robot instead. Participants also noted that daily housework was extremely energy-depleting, and they preferred to use their limited energy on more meaningful activities.

Several participants mentioned that they desired assistance with transfers. This includes being assisted from the wheelchair to the bed or vice versa, as well as being helped up from the floor after a fall or receiving assistance with minor positioning corrections in their wheelchair. Assisted transfers were tasks the participants needed help with several times during the day. This assistance facilitated participation in chosen activities and also prevented the resurgence of pressure ulcers. One participant expressed: *“I imagine that it [a robot] uses its arm, and then I hang on the arm, yeah, just lifts me, and that’s how I imagine it, and then he [a robot] goes over to the chair and then like gently puts med down […]. That would be nice”. (F1-2).*

Most participants said it would be useful if a robot could bring them things and pick up objects from the floor. They mentioned that being assisted in tasks such as bringing a glass of water, providing food, retrieving clothes, or retrieving items they had dropped on the floor would all be helpful. Some mentioned that this could also relieve their informal caregivers of some of these recurring daily assistance tasks. One participant stated:

but it would have at least helped my wife a lot (getting assistance from a robot), that’s for sure, because she brings me different things, like this and that, and even if I lose something on the floor, I need help to pick it up. Things like that would have been great help for me anyway”. (F2-4).

Assisting participants in physical exercise was identified as a need in the groups. Some of the participants in wheelchairs with impaired hand function mentioned the need for assistance at the gym, particularly with attaching to the gym equipment. They needed special gloves to hold onto the equipment but could not manage to put on the gloves and connect to the equipment themselves. Others expressed the need for a walking partner to assist with pushing their wheelchair or as something to hold onto for balance. One participant stated: *“I would like it (a robot) to attach me to different apparatus and assist with the gloves I’m using and support me when we are doing balance training”. (F1-5).*

A few of the participants even said they would not mind if a robot assisted in more personal care tasks such as toileting, showering, or getting dressed. These statements were however most prevalent in the participants who had the least complex needs. One participant stated: “*I would have accepted all the help I could get, yes If he (a robot) could manage it, showering, using the toilet and all those things, that would have been ok”. (F2-4).*

#### The freedom of being alone

The participants depended on help from formal or informal caregivers and many shared the experience of rarely having any time alone, which they missed and found challenging. Although the participants repeatedly expressed appreciation for their assistants, some wished they did not have to constantly interact with the caregivers. Some longed for a “silent caregiver” and desired to be silent themselves, yearning for some time alone. Expressing a desire for “free time,” some of the participants suggested that a robot could enable independence from caregivers for parts of the day.

it’s kind of demanding… living like that… yes almost like having strangers around you at all times….that.... that’s perhaps the nicest thing about something like that (a robot) you could actually not have to deal with other people all the time; (F2-1).

Some participants mentioned that they would occasionally send their caregivers home to spend time with their partners. This meant that their partners had to provide the necessary assistance during that time, but it allowed the couples to have much-appreciated private time. Others reported experiencing a type of social fatigue when their assistants were present. They noted that they expended all their social energy on their assistants and, as a result, would cancel appointments with friends, feeling like they had “nothing left” at the end of the day.

*Can I say something about that. What’s dangerous for me is that I use up.... I am pretty social, therefore, I use up my social “thing” together with my assistants all day. So, in the afternoon…I should have talked to (family and friends)… then I have nothing left. So, in that sense it could maybe be ok with a robot and then I’d have to work on getting in touch with regular people. Because now I’m using up almost all my social “thing” with my assistants. (F3-2).*

#### The ability to decide what to do and when to do it

The participants with the most severe impairments all had personal assistants, but they still spent some periods of the day alone. Many had applied for more hours of care, but their applications were denied. They explained how the assistance services the municipality offered lacked flexibility, preventing the option of being impulsive or participating in activities and society at large. Specifically, all appointments and social engagements had to be planned around the assistant’s presence. Some stated that having a robot that was always present in addition to a human assistant could potentially support their desire to do things on their terms.

One of our participants was born with a physical disability but managed to maintain a full-time position and was actively engaged in organizational work until she suffered a stroke. Her function deteriorated, and she suddenly required full-time assistance and care from her relatives. Despite her cognitive function remaining intact, her carer could not prioritize her desire to continue her organizational work. In her statements, she repeatedly expressed her wish to return to this work. She stated that what she wanted most from a robot was assistance with answering emails and keeping up with the tasks she used to perform (F4-3).

The participant’s ability to control and choose their activities and level of participation became a central theme in their statements. Some individuals expressed a desire for a robot capable of assisting in various tasks, linking this desire to their wish for the freedom to pursue personal preferences at their own discretion.

[…] because… I get assistance to shower once a week, but if I had it (EVE) as well, then I could shower… whenever I wanted. (F4-2).

I agree with (name), I get to shower twice a week, but I would really like four times or even five… (F4-1).

A participant with two limbs amputated struggled with getting dressed and was denied the hours of assistance he felt he needed. His statements reflected his dissatisfaction with the services provided by his municipality. When the participants discussed how a robot could potentially assist in becoming more independent, he shared that he often waited for carers who never showed up. He had to cancel plans some days as he could not leave the house alone.

Other participants shared that waiting had become a part of their new reality and that waiting often dominated their days. This included waiting for home nurses to arrive, waiting for someone to assist when their regular carers were unavailable, or waiting for their spouse to come home to help with tasks they could easily do before becoming disabled. One participant expressed: *“Well- then I just have to wait; I’m good at waiting; if there was a world championship in waiting, my chances would be really good”. (F2-1).* He emphasized the benefits of having a robot on hand to address immediate needs.

The feeling of being a burden was prominent in most interviews, especially when receiving help from their next of kin. Some of the participants expressed feeling uncomfortable when they had to ask for assistance frequently. They often considered whether they needed help or not. Some participants even felt uncomfortable when being assisted by their assistants. While assistants are hired to help with a variety of personal care or practical tasks.There were certain tasks that participants would not ask for assistance with. They believed that these task could be considered boring. One expressed:

I would like a robot to scratch me. I hardly ever ask my assistants to scratch me, then I think of something else, till it eventually stops itching- and right […] and if you had a bowl of chips, you don’t really bother to ask your assistant, can I have one more, can I have one more, but you could do that with a robot. (F3-3).

Some participants felt that as employers they should contribute to making their assistants’ day more interesting or fulfilling. Two participants expressed feeling like they were at work themselves as soon as their assistants arrived, finding it difficult to relax and focus on their activities. They felt constant pressure to fill their assistant’s days with meaningful tasks.

I feel that… when my assistant is there, and I go into my office to work […], the assistant just sits there. That just doesn’t feel right- it’s not easy. But if I had a robot, I wouldn’t have given a damn about it. (F3-1).

### Appearance and functionality

During the discussion on the potential usefulness of the example robot, EVE, the participants shared their opinions on its appearance and functionality. Most participants, especially those with the most comprehensive impairments, admired EVE’s appearance, as seen in the videos. They preferred that the robot’s appearance resembled a human to some extent, mainly for functional reasons but also for considerations regarding the robot’s presence in their homes and how visitors would react to it. Based on its appearance, participants expected EVE to perform tasks similar to humans, despite understanding that its functionality was not yet at the same level as humans. However, they did not favor making EVE look even more human-like or pretending to be human, as they found it weird or uncomfortable.

I think it’s really good that it’s like a human; then it will be able to do tasks more like a human. (F1-3).

When you have visitors, and there’s a huge black box there… well, that’s not as nice as seeing robot that’s a bit human-like. (F1-2).

Well, it will never be a human, so I think it’s important that it looks like a robot. In a way that makes it more realistic […] but still try to be anything else than it is… a robot. (F3-4).

I think people visiting will get shocked no matter what. (F1-1).

During the discussion, participants suggested that a robot’s voice should be customizable to fit each user’s preference, given their familiarity with smartphones and other technology with similar capabilities. They expressed interest in communicating with the robot by issuing commands but not necessarily engaging in deeper conversations.

No, I wouldn’t have any deep conversations with it just yet; it depends; it’s probably more about making it sing some lullabies and simple things like that. (F3-2).

Although the participants appreciated the robot’s ability to reach higher levels, they all agreed that EVE appeared too large. As wheelchair users, the notion of a robot looking down on or asserting dominance was undesirable. Therefore, a suggested solution was a smaller and more maneuverable robot, especially suitable for smaller apartments. Despite a smaller size, an elevation function allowing it to reach higher shelves and closets was recommended. When asked about their willingness to try a robot in their home, even in its incomplete state, all participants expressed interest in exploring its potential usefulness.

### Concerns about having a robot as an assistant

The third theme identified the participants’ concerns when they imagined having a robot as their assistant. Although all 18 participants agreed that receiving assistance from a robot was acceptable in some ways, there were certain tasks they would not allow a robot to help with, mainly those involving personal care, such as assisting with showers, toileting, and transfers (refer to Table 3). They attributed this reluctance to a general distrust or skepticism about the robot’s abilities. The participants’ concerns also revolved around the robot’s size, placement, privacy issues, and lack of human contact.

The participants’ concerns about a robot’s functionality were evident in their explanations of the complexity of their needs. Some participants with spinal cord injuries required assistance with urinary catheterization and stimulating bowel movements, which carers performed while communicating closely with the users. These procedures often varied slightly from day to day. Participants expressed that they would not trust a robot to perform these tasks due to the inability to communicate with the robot about the necessary details and a concern that the robot could cause harm to their intimate areas. Similarly, some participants believed that a robot could not assist with transfers without exposing them to harm, such as falls or pressure ulcers. However, it was noted that while they had these concerns, they were optimistic about the possibility of robots performing such tasks in the future.

I’m not sure what it would be like, but I think it’s a while until it’s gonna be like useful for anything at all. These tasks sound simple, but it’s still complex anyway. (F2-1).

if my assistants did not come to work, I would still have to have a human present for catheterization and things like that… I don’t think it could manage doing that […] it would be a bit difficult to teach it- but maybe in twenty years it’s much more developed. (F1-3).

A participant, who was largely independent in most activities but required assistance with lifting heavy objects and complex fine motor activities, noted that: *“I can’t really see… when it comes to my needs. It is the more advanced things I need assistance in”. (F2-5).*

During the study, participants were informed about various ways of controlling robots, such as using VR glasses, an app, a joystick or buttons, voice commands, or remote control from a central unit in the municipality where health personnel could operate it. Some participants found controlling a robot via an app, buttons, or VR glasses unacceptable due to their lack of hand function. The majority preferred voice commands, but one participant expressed concern that a robot may not understand the voice commands at night when wearing a breathing-assisting mask. While remote assistance was accepted if no other options were available or in emergencies, most participants were hesitant about someone else looking into their homes through a robot’s “eyes.” Two participants expressed discomfort, stating: *“Knowing that someone else were controlling it like that… I would not have liked it”. (F2-1). “I think it would have been creepy… that I didn’t know who’s looking. I wouldn’t like it”. (F3-4).* One participant had a more creative suggestion:

I would have wanted my [human] assistants to have “passive evening shifts” at home [controlling a robot], then they wouldn’t have to go out to assist me, and I would get assisted by someone who knew me. (F3-2).

Although the participants did not emphasize the lack of human contact as a major concern, they discussed how the presence of a robotic helper could potentially replace human assistance and lead to a desire for human contact and social interactions, not just for themselves but also for others.

I’m very much like…I have a very social job, so for me, not having people around at home can be nice sometimes […]. But then I think of those who are disabled or something like that, they are home a lot of the time, losing that human contact, and that is something that humans need…, and if you don’t have a particularly large social network […]. Well, maybe a combination would be ok. (F3-4).

One participant expressed concern about the potential loneliness of having robotic assistants, noting that he would miss the conversations and jokes he shares with his human home nurses during morning care. He stated, *“I have to say it’s kind of nice when the home care nurses arrive in the morning; we can talk about all kinds of nonsense”. (F3-1).*

## Discussion

This study investigates the types of assistance tasks that people with physical disabilities would accept from a robot and their perceptions of having a robotic assistant in their home. The participants expressed finding robotic assistance useful and acceptable for a range of tasks, but their need for such assistance was closely related to their level of functional disability. For instance, participants facing the most significant challenges in ADL and requiring the highest level of assistance, expressed a stronger preference for robotic assistance. Furthermore, their satisfaction with their current care situations might have influenced their perceptions of whether they would accept robotic care as a future healthcare service option. Our participants agreed that household tasks and servant-like functions were acceptable to be received by a robot. These findings align with studies investigating older adults’ attitudes and preferences for robotic assistance [29, 46, 47]. Some of our participants with mobility loss mentioned that being assisted in transfers at times of their choosing would contribute to a feeling of personal autonomy and independence, including after a fall. Similar findings were reported by Beer et al. (2015) in a group of older adults with mobility loss [46]. However, some of our participants were skeptical about a robot’s ability to perform such assistance safely, doubting that a robot would assist with transfers.

In contrast to several studies involving older adults, some participants were willing to accept and appreciate assistance in personal care, such as dressing, feeding, and even toileting and showering [48, 49]. However, the statements about toileting and showering were more prevalent among participants who did not have the most complex needs. Participants who had no skin sensation due to paralysis and those who required catheterizations would not accept robot assistance for these tasks under any circumstances. Nonetheless, they were more open to robotic assistance for dressing and feeding tasks. These findings are consistent with studies involving similar participants [50–52].

Assistive technologies have made tremendous progress in recent years, and we expect this trend to continue. Some of the tasks that participants in our study found useful, such as bringing food and drinks and picking up items from the floor, have been tested by autonomous robots in previous studies [53–55]. Transfers have also been tested using the Japanese robot Riba [56], and personal care tasks such as feeding and washing the face have been investigated in clinical studies using the PR2 robot [57, 58]. However, our participants had complex needs, so robotic assistance for these tasks must be tailored to each user’s preferences and context. Despite these advancements, robots that assist with various physical tasks have not yet been commercialized or implemented in users’ homes.

Our findings indicate that the participants were hesitant about the prospect of assistive robots being remotely controlled due to privacy concerns. However, they were willing to accept such assistance if it supported their needs and addressed privacy concerns. One of our participants suggested that a home nurse or assistant controlling a robot from a distance could be the first step in assisting users with some tasks that could promote their autonomy and independence.

The participants discussed whether a robot should look more or less human-like. Most participants found the robot EVE’s appearance acceptable, and they expressed discomfort with the idea of a more human-looking or behaving robot. This feeling of eeriness has been described and named the “Uncanny Valley” by Mori (1970) [59]. Mori observed that robots appeal more to people with a human-like appearance, but only up to a certain point [59, 60]. After reaching the uncanny valley, a person’s affinity decreases into a feeling of unease and a tendency to become scared. All participants mentioned that the current size of EVE was too large, but they appreciated its ability to reach objects at higher levels.

The participants held mixed views on how to control a robot. Although most preferred a voice-controlled robot or a mix of tablet and voice control, concerns were raised about whether a robot would understand voice commands, especially by the participant who used a breathing mask at night. Other studies have also reported mixed feedback regarding robot control, which could be related to users having different levels of impairments and prior technological experience [61, 62]. Although we did not specifically assess this, we found that most of our study participants had extensive experience using assistive technologies. They mentioned using electric wheelchairs, smart house functions, and adapted cars, with different control and interfacing options. This experience might have influenced their perceived ease of use and positive attitudes toward robotic assistance [26, 27].

It was evident that the positive perceptions of these participants regarding assistance from a robot were linked to their desire for more autonomy, empowerment, and greater independence in their daily activities. The participants were not afraid to use new technologies or concerned about any stigma associated with assistive technology. All the participants had experienced significant changes in their ability to perform essential occupations, which affected their independence. Engaging in meaningful activities or occupations positively impacts health and well-being [63, 64]. When individuals with physical disabilities can no longer carry out these occupations, they may lose their roles and have their routines interrupted [63, 64]. Several of our participants felt like a burden, primarily towards their family members and their formal carers (UPAs). Ch`ng et al. (2008) [65] found that for new stroke sufferers, loss of social roles and reliance on others, suggesting a sense of burden, were linked to depression and suicidal ideation. Feeling like a burden is a distressing sensation associated with self-perception, quality of life, and loss of autonomy [66].

Many participants wanted to be alone, but their physical limitations made it impossible. Robotic assistance was discussed by the participants as a potential solution, replacing human caregivers and facilitating moments of quiet and quality time with partners. This differs somewhat from research on older adults, which found that robots stimulated conversation and prevented feelings of loneliness [67].

In this study, we invited potential future users of robotic assistance to share their perceptions of a new way of delivering healthcare services in the home. Since the presentation of the “Innovation in Care” report (2011) in Norway, several municipalities have experimented with welfare technologies in users’ homes, mostly for the older population, but not all have been successful. The success rate can be attributed to both the user’s need for the specific technology and its usability, and the organizational structure involved in providing it [26, 27]. According to the literature, a person’s perceived need for certain robotic assistance and ability to use the robot predict their intention to use it in the future [27].

Norway’s health services are widely regarded as among the best in the world [68]. However, our participants’ stories reveal they lack the freedom to decide or act according to their self-chosen wishes and plans. The participants receive assistance from various service providers, including informal carers, assistants, or home nurses. While most participants appreciate the services they receive, they welcome personalized robotic assistance for various tasks, as it would contribute to their sense of autonomy and independence. They view robotic care as additional help that can fill gaps when they do not have a human helper. Furthermore, they see it as a way to give their carers a break and enjoy some private time without needing to communicate or socialize, which felt required in their human carers’ presence.

The findings of this study provide useful insights for developers of complex assistive technologies and stakeholders involved in planning and providing healthcare services in municipalities. Our study contributes to the existing literature on accepting robotic assistance in users’ homes by providing insights from a population of users with physical disabilities.

### Methodological strengths and limitations

The focus group interviews provided a comfortable and permissive environment for the participants to share their perceptions. The researchers created a safe space, and the participants were talkative and respectful toward each other. The researchers’ background in health occupations enabled them to provide the necessary assistance during the sessions and understand the participants’ medical and professional terms.

To ensure the trustworthiness of our results, we took several steps. We pilot-tested the questionnaire to ensure the questions were appropriate and easily understood. We involved end-users in the design of the data collection process. During the interviews, we listened carefully to the participants and sought clarification on areas of ambiguity. Finally, we asked the participants to verify our summary comments immediately after the interviews [35].

Three participants who were outside the inclusion criteria were recruited for the study. This may have been due to the coordinators’ busy schedules and lack of follow-up questions when providing information. Two of these participants that were over 70 years old were still included, as they lived at home and led active lives. The third participant that was in a group with dropouts had severe aphasia, leaving this group with only two participants. Consequently, the moderator could only pose closed and somewhat leading questions, leading us to eventually exclude the results from this group, potentially impacting the overall findings. Several recruited participants failed to attend at the last minute, a common challenge in similar groups. Tausch et al. [37] recommended over-recruiting to focus groups to address this issue.

We aimed to accommodate for participation by letting potential participants choose from dates and times. This impacted on the composition of the groups in terms of types of disability and sex.

The use of purposive sampling may have introduced bias in terms of including participants who found the topic of robotic assistance exciting, potentially influencing the relatively positive attitudes towards robotic assistance. To accommodate for participants’ needs, we arranged one of the focus groups on a digital platform, which could affect group dynamics as there was no opportunity for small talk or a light snack as in the other groups. Moreover, the participants in this group watched movies of the example robot EVE before the interview, which may have influenced their perceptions of robotic assistance.

We acknowledge that the small sample size of 18 participants may be a limitation. However, we believe that the information power was sufficient. The small but rich group of participants had physical disabilities and was dependent on assistance to varying degrees. The participants were talkative and generously shared their life experiences and perceptions of a robot as a possible new healthcare service. As suggested by Malterud et al. [38], information power is reached when participants generously share their experiences so that the aim of the study is achieved. The information power of our study was further strengthened by the rigorous six-step analysis that resulted in new and nuanced patterns relevant to the study’s research questions and aims [34, 38].

A significant portion of the population experienced Spinal Cord Injury, which could have potentially biased the results. Nevertheless, the expressed needs in our findings are remarkably consistent across various types of disabilities, emphasizing functionality rather than the specific nature of the disability, such as reduced upper limb function.

Furthermore, the sample comprised a greater number of male participants compared to females, reflecting the population affected by stroke and spinal cord injuries [69, 70]. Despite this disparity, no discernible differences were observed in terms of needs and acceptance between males and females.

## Conclusion

Our participants with physical disabilities perceived robotic assistance as acceptable for various tasks. They welcomed the idea of a future robotic assistant in their homes for areas that could contribute to increased independence and autonomy. In some areas, the participants would consider replacing a human helper with a robot; in others, they saw a robot as a supplement to their human assistance. However, for a robot to be useful, it would need to be individually customized to meet the needs of each user, both in terms of which tasks it can assist with, how it can assist with these defined tasks, and how it is controlled.

### Electronic supplementary material

Below is the link to the electronic supplementary material.

Supplementary Material 1

## Data Availability

The data generated and analyzed during this study are not publicly available due to concerns about confidentiality regarding a small sample size and the sensitive nature of the interviews. Instead, quotations and analytical categories are included in the text. We are happy to discuss the findings or the analysis if any questions arise.
