# Supplementary material for: Care-receivers with physical disabilities’ perceptions on having humanoid assistive robots as assistants: a qualitative study
Source: BMC Health Serv Res. 2024 Apr 25;24:523. doi: 10.1186/s12913-024-10857-9 (PMC11044328; doi:10.1186/s12913-024-10857-9)
Supplement: Supplementary file 1 — Supplementary Material 1 [file 12913_2024_10857_MOESM1_ESM.pdf]

**Interview guide, focus group semi structured interview**

| | |
|---|---|
| Research question: **Which service tasks would care-receivers with physical disabilities` accept or not accept to be delivered by a robot?**<br>**What are their perceptions on receiving robot assistance in their homes?** | |
| The participants receive information about the project, their rights, the time frame as well as practical information, and get the opportunity to ask questions. | |
| The participants fill out a form with name, age, diagnosis, and tick off activities they receive help in and how often. | |
| **Opening questions** (goal: getting everyone to talk effortlessly throughout the interview, this will not be analyzed) | 1. Tell us your name, age and what you do on a daily basis. (Round) |
| **Introductory questions** | 2. "In this group, there are many different experiences with assistance in daily activities that it would be interesting to hear a little more about. Will one of you start by telling a little about these experiences and about the help you receive."<br>3. How often do you get help with this? |
| **Transitional issues** | 4. Are there any tasks you don't get assistance in today that you would like to get help with? |
| **FILM WITH DEMONSTRATION OF EVE THE ROBOT** | |
| **Key questions** | 5. If we imagine that it will not be possible to get as much help as today-<br>Imagine that a robot, ie EVE was one of the services who could help you. If any, what kind of tasks do you see a robot helping you with?<br>6. What kind of robot assistance could have an impact on your independence?<br>7. Are there any tasks you would prefer getting help from a robot rather than a human?<br>8. What tasks wouldn't you let a robot help with, and why?<br>9. You've told us a little bit about getting help with ...., are there any things you wish you could get help with right away/that it is challenging to wait to get help for?<br>10. What do you think about the fact that a robot resembles a human being?<br>11. What do you think are the practical limitations or disadvantages of having a robot at home? Examples |
| **Concluding Questions** | 12. We have now talked about... . is there anything else you'd like to add about this that we haven't touched on, or that you haven't been able to say so far? |
| Summarize and request verification | |

*Questioning route* after Kruger and Casey 2015.
